# Cause Analysis and Preventive Measures of Guizhou D2809 Train Derailment Accident in Guizhou, China on 4 June 2022

**DOI:** 10.3390/ijerph192417003

**Published:** 2022-12-18

**Authors:** Yan-Ning Wang, Han Chen, Bin-Song Jiang, Jing-Rui Peng, Jun Chen

**Affiliations:** 1 MOE Key Laboratory of Intelligence Manufacturing Technology, Department of Civil and Environmental Engineering, Shantou University, Shantou 515063, China; 2 State Key Laboratory for Geomechanics and Deep Underground Engineering, China University of Mining and Technology, Xuzhou 221116, China; 3 Department of Civil Engineering, Shanghai Jiao Tong University, Shanghai 200030, China

**Keywords:** train derailment, debris flow, accident rescue, prevention and control, Guizhou

## Abstract

This paper summarizes the cause of the debris flow impact train accident by investigating the local geological condition, meteorological data and field investigation that happened in Guizhou, China on 4 June 2022. The result showed that the major reason is the continuous heavy rain in the surrounding area, which led to a small landslide at the upper right of the tunnel entrance. Besides, the construction of the Jianrong Expressway in the upper reaches increased the catchment area, which makes the water content of the upper soil increase while the shear strength decreases. Such large-scale catastrophic accidents significantly threaten the local environment and public safety. Therefore, it is urgent to pay special attention to the changes in geological conditions along the line, especially the adverse effects of construction, to improve the early risk warning and post-accident treatment ability.

## 1. Introduction

The occurrence of major traffic accidents seriously affects public safety. The derailment factors caused by different types are shown in Table 1 in detail. The derailment of the Jiaoji line on 28 April 2008, as the speed exceeded the safety limit, caused 72 deaths and more than 100 injuries [1]. The derailment of Amtrak Passenger Train 188 Philadelphia, Pennsylvania, 2015 due to excessive speed when crossing the curve, caused eight deaths and more than 193 injuries [2]. The train derailment on 30 March 2020 due to a sudden landslide, caused one death, four serious injuries, and 123 minor injuries [3]. This derailment of the D2809 passenger train was an accident on a debris flow-prone section of Guizhou Province during heavy rainfall, leaving one dead and twelve others injured.

As a serious public safety problem, train derailment accidents have attracted wide attention from scholars [4]. Zhai et al. developed an intelligent analysis support system (IDASS), which provided an information analysis method for the investigation and analysis of derailment accidents [5]. Liu et al. developed a zero-truncated negative binomial (ZTNB) regression model to estimate the conditional mean of train derailment severity [6]. Wu et al. studied the dynamic characteristics of a high-speed train after derailment under earthquake excitation and established the mechanical model of a high-speed train after derailment [7]. Ling et al. presented the formulation of a train–track–bridge interaction model to illustrate the collision induced derailment mechanisms of a passenger train traveling on a box girder bridge [8]. However, there is still a lack of relevant research on train derailments caused by debris flow in mountainous areas.

It is well known that China is one of the regions with the longest operating mileage of a high-speed railway and the largest number of passengers. The safe operation of a high-speed railway is critical to the safety of the public. For a long time, China’s high-speed railway has maintained a good safety operation record until the safety incident mentioned in this article occurred. Therefore, this paper reports the detailed process of this event and makes a preliminary analysis of the causes, which we believe will help to arouse relevant research interest or improve the research level of relevant fields.

**Table 1:** Factors of train derailment [9].

Major Factor	Sub Factors	Description
Poor track condition	Expanding rail track Gauge widening Broken rail Pier failure Switch failure	Heating expansion Abnormal stress Circuit fault Switch circuit failure Subgrade frost heave
Speeding	Overspeed crossing Curve of speeding	Over speed of bifurcation/curve section
Natural hazard	Strong wind Snowstorm Landslides	Wind and snow interference train Foreign body invasion
Train	Poor operating condition Brake failure Train partial load Tailgating Slippery	Abnormal train operation Abnormal train braking Partial load of train Train rear-end collision Train sliding
Else	Human error, etc.	Stealing rails Damaging cables, etc.

## 2. Accident Process and Rescue Measures

### 2.1. Accident Process

**Figure 1:**
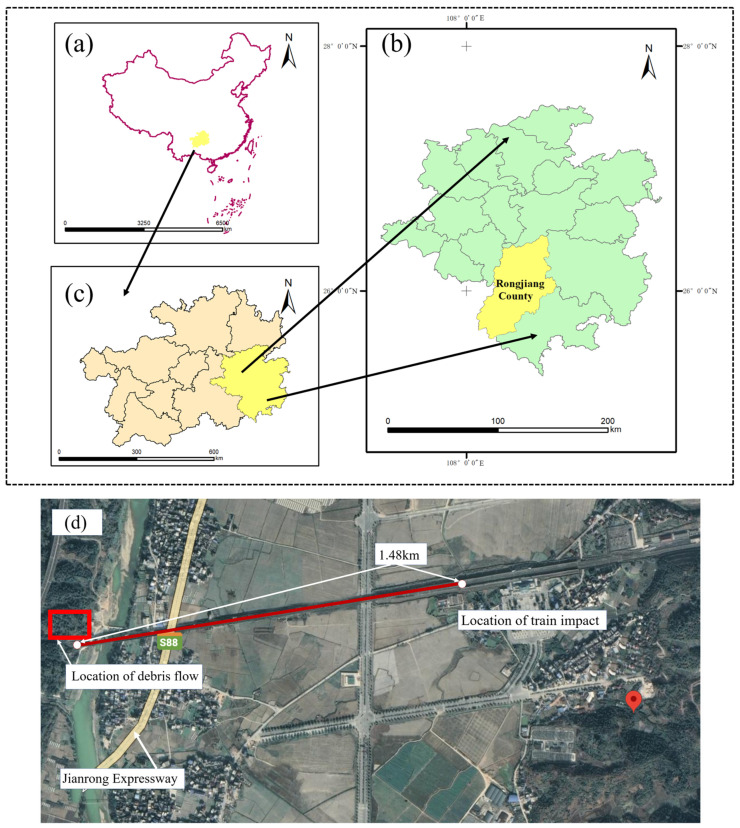
Location of the accident site: (**a**) Guizhou Province in China; (**b**) Qiandongnan Miaodong autonomous prefecture; (**c**) The disaster site in Rongjiang County, Guizhou Province; (**d**) Satellite image of the accident site.

According to the official news of the Southwest Railway, at around 10:30 on 4 June, Bullet train D2809 departing from Guiyang to Guangzhou came off the tracks in Rongjiang county, leaving one train driver dead and 12 others injured. Among the injured were two members of the crew and 10 passengers. The train hit mud and rockslide debris which had suddenly fallen onto the tracks, causing two carriages to come off the tracks. The location of the accident scene is shown in Figure 1, about 1.48 km from the collision tunnel.

**Figure 2:**
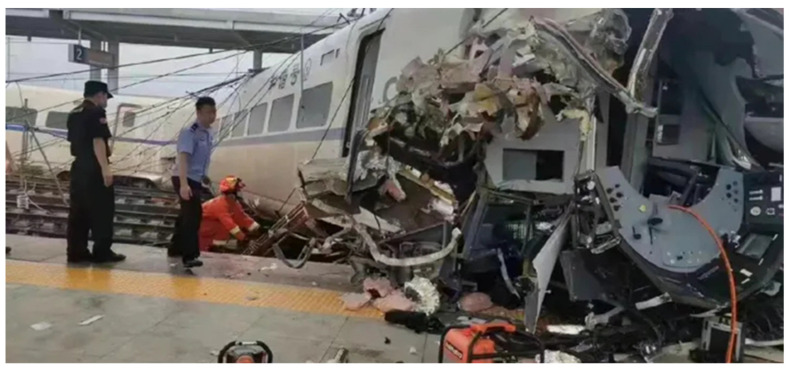
Accident scene (Source: https://www.163.com/dy/article/H9PN38TB0552ZFBN.html, accessed on 4 June 2022).

A small landslide occurred at the top right of the tunnel entrance of the bridge across the Zhaizhajiang River in the direction of Guiyang, and a debris flow body invaded the main line of the Guigang Passenger Line. On the bridge across the Zhaihao River, some power supplies hit the net. A bullet train rushed through the mud pile on the tracks and crashed into the platform of Rongjiang Station [10]. Figure 2 shows the damage of the high-speed train in detail; it can be seen that the front of the train is seriously damaged. 

The accident occurred near Rongjiang Station in Guizhou Province. Rongjiang Station, located in Rongjiang County, Guizhou Province, China, is a third-class station under the jurisdiction of China Railway Chengdu Bureau Group Co., Ltd. This station started construction on 30 December 2013, underwent the trial operation in August 2014, and was officially completed and delivered on 26 December 2014.

### 2.2. Rescue Operations

**Figure 3:**
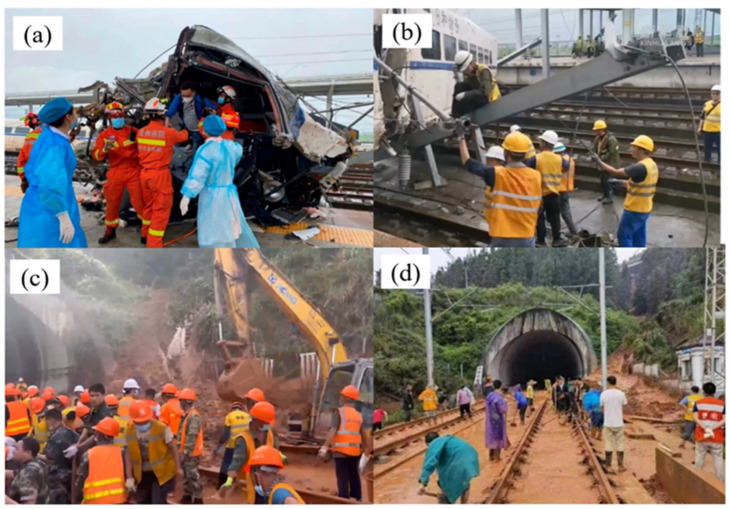
Rescue operation: (**a**) Firefighters rescuing trapped people; (**b**) Staff cleaning up the accident train; (**c**) Track debris flow removal; (**d**) Clearing debris flow intruding into the line (Source: Xinhua News Agency https://haokan.baidu.com/v?pd=wisenatural&vid=2796812779194810025, accessed on 24 June 2022).

Immediately after the accident, the railroad department started the emergency plan, the injured were sent to Rongjiang County Hospital for complete treatment and the local fire department immediately sent two heavy rescue teams totaling 52 people and 11 cars to the scene for rescue. Injured passengers and train crew were appropriately treated, with no life-threatening. The driver of the train unfortunately died. The other 136 passengers on the train were arranged to transfer to evacuation. During this period, the scene rescue work was fully launched, and the cause of the accident was investigated. The rescue actions taken by the rescue team at the accident site is shown in Figure 3. Firefighters and medical personnel rescued the wounded trapped in the train, maintenance personnel cleaned up the wreckage of the accident train, and people cleaned up the debris flow on the railway tracks [11].

## 3. Analysis and Discussion

### 3.1. Cause Analysis

**Figure 4:**
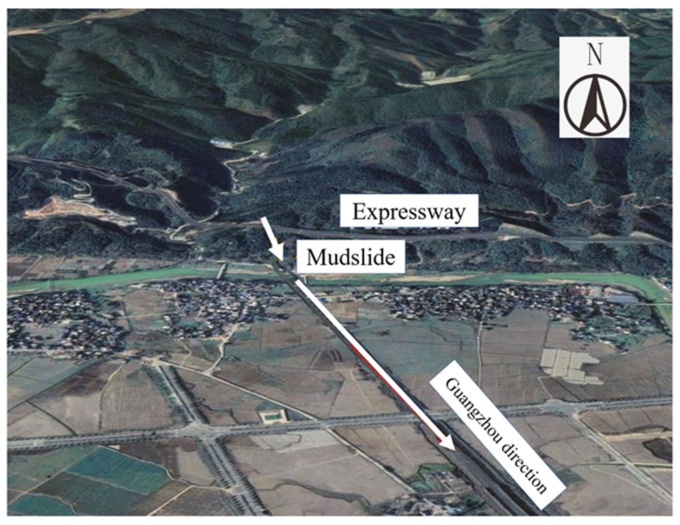
Accident simulation analysis diagram.

According to preliminary analysis, the accident is mainly due to continuous rain and short bursts of heavy rain in the area. The high-speed train suddenly slipped into a landslide on the line, causing cars 7 and 8 to derail. The accident simulation analysis diagram is shown in Figure 4. According to the data analysis on board, the driver on duty found that the line abnormality train was traveling in the former Yuezhai tunnel at Rongjiang Station. The train skidded more than 900 m in less than 5 s when it made an emergency brake, greatly reducing the collision speed, and avoiding a more serious tragedy. Human error was ruled out as the cause of the accident. At the same time, the anti-collision wall of the high-speed railway and the overall protection of the track structure prevented the train from overturning and falling. However, the formation mechanism and disaster characteristics of this debris flow still required further investigation and analysis.

### 3.2. Causes of Debris Flow

**Figure 5:**
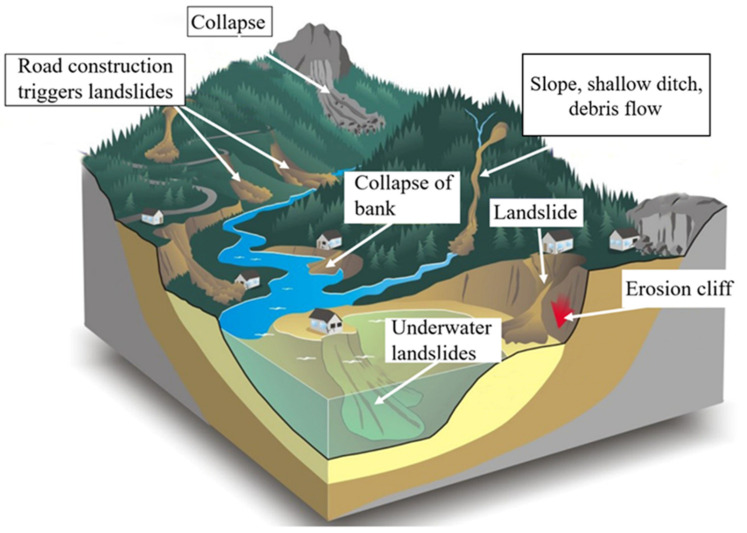
Different types of debris flow under different terrain conditions.

Debris flow is a natural phenomenon, usually originated from landslides, with high impact force, characterized by no signs and rapid movement [12]. Generally, debris flow is characterized by rapid movement, sudden occurrence, fluid-like movements and long travel distances from their outlet. Some studies indicate that mudflows are triggered mainly by excess pore pressure or liquefaction processes in the material. The dynamic stress peak determines the deformation mode of the elastic-plastic plate model. The impact failure behavior of coal and rock is the most obvious [13], generating sudden and high losses in shear resistance, generating a flow of mud [14,15]. Classification of debris flow according to landform is the most commonly used method. The measurement of elastic strain energy (ESE) of rock joints is an essential basis for evaluating the shear strength of rock mass. Joint morphology and rock mass strength are considered vital factors affecting ESE distribution [16]. Whether in a case study or the study of a regional characteristic, many experts and scholars have determined types of debris flows according to the geomorphological position or gully geomorphology. Furthermore, most of them have adopted dichotomy to divide debris flows into gully debris flows and slope debris flows [17,18,19,20]. Guizhou Province is located in the middle and low latitudes, on the eastern slope of the Yunnan-Guizhou plateau. After a flash flood, rainwater will bring loose materials to both sides of the gully, flow into the gully bed and then flow into the river in large quantities. It is straightforward to cause flash floods and mud rock flows [21]. According to the survey, hundreds of extensive landslides occur yearly during the rainy season across the province, producing different debris flows. Figure 5 shows different types of debris flows under different terrain conditions.

The dynamic changes of geological disaster-prone areas around faults and rivers can be attributed to earthquakes and precipitation intensity changes in different periods [22]. The exposed strata in this area include the Fanzhao Formation (Qbf), Qingshuijiang Formation (Qbq), Pinglue Formation (Qbp), Cretaceous Maotai Formation (K2m) of Qingbaikou System and Quaternary [23]. The Cretaceous Maotai Formation near Rongjiang Station consists of mudstone in the upper part and gravel in the lower part. The upper claystone is of low strength. Claystone is particularly prone to weathering and is easily softened by water. The conglomerate is a rock that used to be broken by external forces in nature and then formed by consolidation into rock.

**Figure 6:**
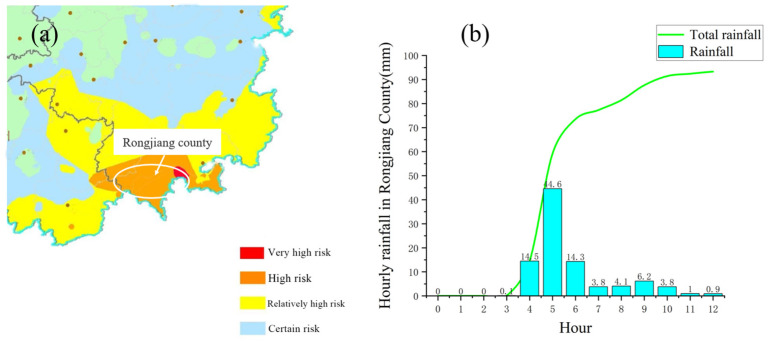
Rainfall situation: (**a**) Rain warning map of Rongjiang County the day before the accident; (**b**) Rainfall in Rongjiang County within 12 h by time.

Based on various information, the reasons for the occurrence of local debris flow are as follows:

(1) Landslide debris flow. Rongjiang County, where the incident occurred, had issued seven weather warnings since the early hours of the morning, including a red warning for heavy rain. The red rainstorm warning issued on the same day and the rainfall curve within 12 h is shown in Figure 6.

According to the weather station monitoring, from 8:00 on 1 June to 8:00 on 4 June, Guizhou Rongjiang and the other nine counties (districts) in the domain of 13 stations, 59 h of cumulative rainfall of more than 300 mm occurred. Landslide liquefaction can be divided into three stages, the compaction of the sand layer caused by the sliding of the upper slope, excess pore water pressure in the saturated area and rapid shear [24]. After heavy rain, the soil moisture content increases, leading to a decrease in soil shear strength. The rapid rise of temperature after rainfall stops, which makes the ground dry and cracked. The stress state of different soil layers changes, which is more likely to induce debris flow.

**Figure 7:**
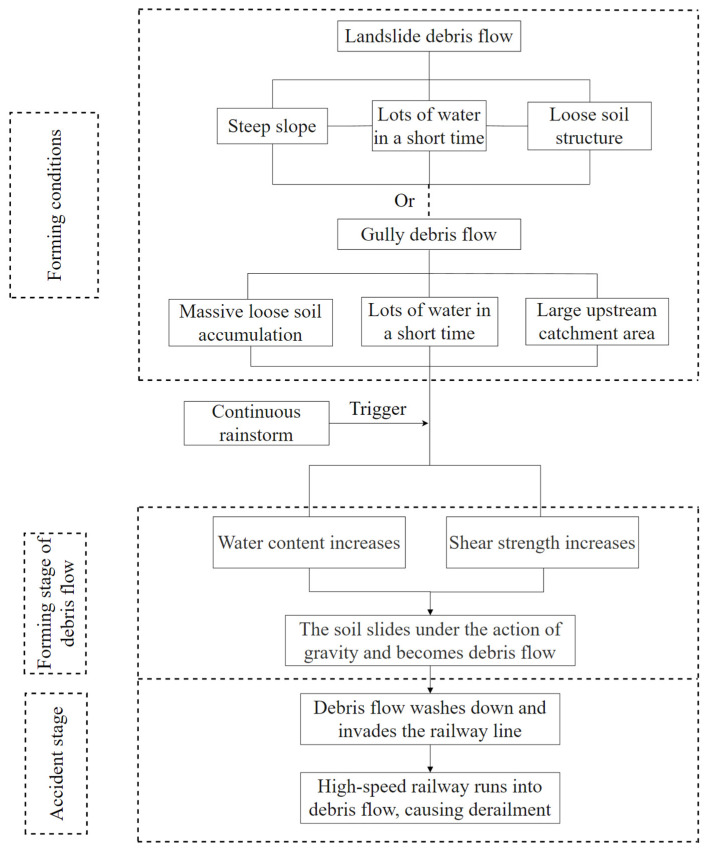
Logic diagram of debris flow analysis and judgment that caused this derailment accident.

(2) Gully debris flow. There are generally three types of gully mudflow: source flow conditions, a large number of loose solid material in the mountain; water conditions, short periods of heavy rainfall or ice melting; and topographic conditions, large catchment area upstream. Before the accident, there was a large amount of earth accumulation at the construction site above the tunnel of the Jianrong Expressway. The continuous rainstorm significantly increased the catchment area, and the earth accumulation upstream became loose. The earth piled up by the roadside of the expressway washed down the railway trunk along the valley, leading to the accident. Based on the above two causes, the logical analysis diagram of the train derailment accident is shown in Figure 7.

### 3.3. Recommendation

**Figure 8:**
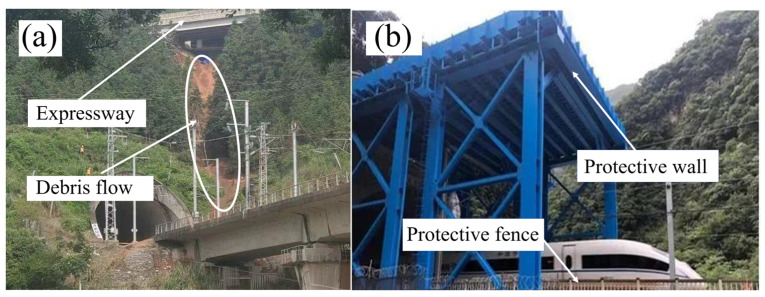
Safeguard: (**a**) Rongjiang Station tunnel exit; (**b**) protective facilities for Xi’an Chengdu Railway Construction to prevent rockfall.

**Figure 9:**
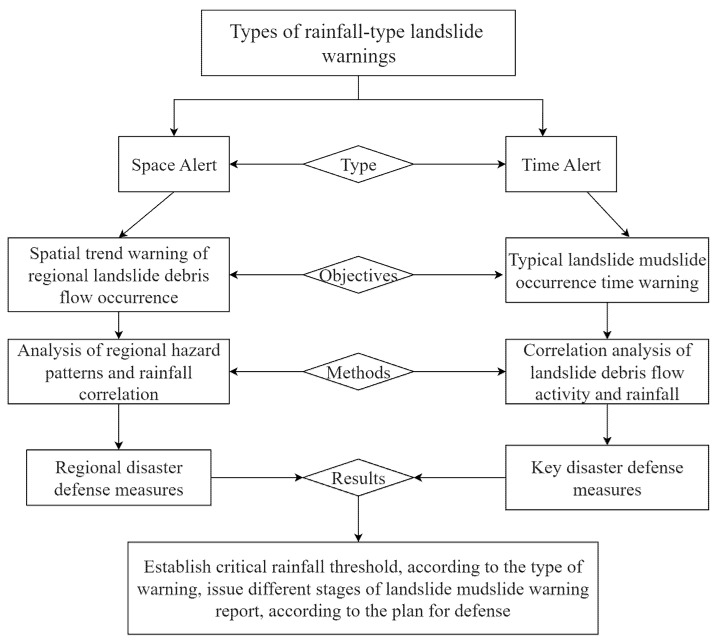
Flow chart of landslide debris flow warning.

The driver of D2809 braked decisively to reduce the speed when the incident occurred. However, the head of the train hit the station platform, and the cab was seriously damaged. The driver died in the line of duty. Without this crucial step, high-speed trains traveling up to 250 km/h might even break through the bridge between the tunnel and the station and crash into the river, causing more casualties. However, in the face of natural disasters, it is the fundamental policy to use technical means to prevent and solve natural disasters as much as possible. Based on the comparison results, the following points are suggested here:

(1) The slope of the mountain where the tunnel entrance is located is enormous, and Guizhou is a region with frequent debris flows. The failure to set a protective retaining wall at the entrance of the tunnel during construction is the main cause of this accident. Therefore, we propose to install a protective retaining wall at the entrance of the tunnel, which is prone to landslide and rockfall, to guide the debris flow or rockfall to the outside of the track and ensure the safety of the railway. In addition, guardrails should be installed along railway lines to alert the police if they break automatically. The Westside Railway, for example, has installed protective equipment to prevent rockfalls. The effect diagram after adding the protective structure is shown in Figure 8.

(2) Floods caused by a large amount of sudden local rainfall, usually accompanied by rapid runoff and debris flow, rise quickly with little or no warning [25]. The debris flow was so sudden that only by human inspection, local departments can not find problems in time.The debris flow was so sudden that only by human inspection can local departments find problems in time. Cameras should be installed at bridges and tunnels, combining manual inspection with automatic monitoring and alarm. During the flood season, the railroad department should conduct a flood safety inspection of critical areas and lines to survey hidden dangers comprehensively. At the same time, a multi-dimensional dynamic corresponding mechanism [26] from types to objectives to methods to results should be established as shown in Figure 9.

(3) From the perspective of site selection, the slope at the tunnel entrance is too large, and there is the high-speed intersection above it. Therefore, the survey site selection should strictly follow the relevant specifications and design rules. In addition, the tunnel entrance should not be located in landslides, collapses, rock piles or dangerous rock rolling stones. Moreover, the location of the entrance should be reasonably selected to avoid the formation of a high slope and elevation and not be set in a significant bias and seriously wrong geological area. It should avoid low-lying drainage difficulties in the valley.

(4) From the point of view of geotechnical engineering, after the construction is completed, the soil at the tunnel entrance can be reinforced by the labor method to prevent a landslide and collapse. The common ground pre-reinforcement construction methods are usually ground bolt and high-pressure jet grouting [27], sonic grouting, etc.

## 4. Debris Flow Risk Grade Evaluation

### 4.1. Establishment of System Hierarchy

**Figure 10:**
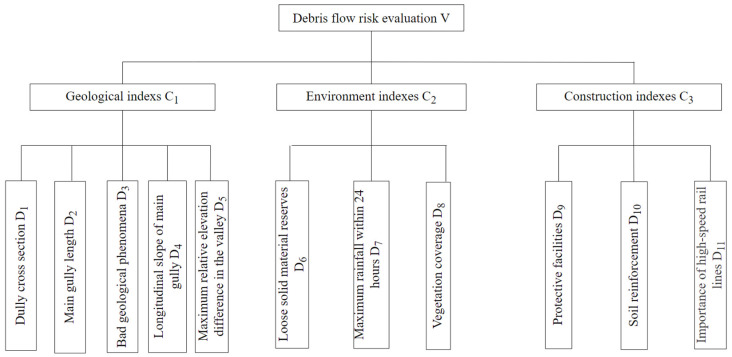
Evaluation index system of debris flow risk in hydropower project.

The risk factors were obtained from other researchers, such as Akgun [28], Wang [29] and Chang [30], and were used to establish the debris flow risk assessment model. All risk factors were selected as follows: dully cross section, main gully length, bad geological phenomena, longitudinal slope of main gully, maximum relative elevation difference in the valley, loose solid material reserves, maximum rainfall within 24 h, vegetation coverage, protective facilities, soil reinforcement, importance of high-speed rail lines. The evaluation index system of debris flow risk in the hydropower project is shown in Figure 10. The corresponding classification standard is listed in Table 2.

The above eleven factors that affect the risk degree of debris flow constitute three first-order factor sets:

(1) $$C_{1}=\left\{D_{1},D_{2},D_{3},D_{4},D_{5}\right\};$$

(2) $$C_{2}=\left\{D_{6},D_{7},D_{8}\right\};$$

(3) $$C_{3}=\left\{D_{9},D_{10},D_{11}\right\}$$

Divide debris flow risk assessment set:

(4) $$V=\left\{\mathrm{Low}\;\mathrm{risk}\;(V_{1}),\;\mathrm{Moderate}\;\mathrm{risk}\;(V_{2}),\;\mathrm{High}\;\mathrm{risk}\;(V_{3}),\;\mathrm{Extreme}\;\mathrm{risk}\;(V_{4})\right\}$$

The analytic hierarchy process (AHP) method is used to calculate the weight of each factor [31]. The weight of each risk factor in the scheme layer can be obtained from Table 3. This method perfectly combines the mathematical model with the expert’s experience judgment and is one of the most widely used weight calculation methods. The weights of each layer in Figure 10 were calculated by the AHP method. The weight calculation results are shown in Table 3.

The consistency of all matrices is acceptable through calculation and verification.

### 4.2. Determination of Membership Function

The membership function *Y*_v1_, *Y*_v2_, *Y*_v3_ and *Y*_v4_ were calculated by the semi trapezoidal distribution model. The equation of the membership functions as follows [32]:

(5) $$Y_{v1}(x_{i})=\left\{ \begin{array}{c} 1,x_{i}\leq e_{1}\\ \frac{e_{2}-x_{i}}{e_{2}-e_{1}},e_{1}<x_{i}\leq e_{2}\\ 0,x_{i}>e_{2} \end{array}\right.$$

(6) $$Y_{v2}\left(x_{i}\right)=\left\{ \begin{array}{c} \;\;\;0,x_{i}\leq e_{1},x_{i}\geq e_{3}\\ \frac{x_{i}-e_{1}}{e_{2}-e_{1}},e_{2}<x_{i}\leq e_{2}\\ \frac{e_{3}-x_{i}}{e_{3}-e_{2}},e_{2}<x_{i}<e_{3} \end{array}\right.$$

(7) $$Y_{v3}\left(x_{i}\right)=\left\{ \begin{array}{c} \;\;\;0,x_{i}\leq e_{2},x_{i}\geq e_{4}\\ \frac{x_{i}-e_{2}}{e_{3}-e_{1}},e_{2}<x_{i}\leq e_{3}\\ \frac{e_{4}-x_{i}}{e_{4}-e_{3}}e_{3}<x_{i}<e_{4} \end{array}\right.$$

(8) $$Y_{v4}\left(x_{i}\right)=\left\{ \begin{array}{c} 0,x_{i}\leq e_{3}\\ \frac{x_{i}-e_{3}}{e_{4}-e_{3}},e_{3}<x_{i}\leq e_{4}\\ 1,e_{4}\leq x_{i} \end{array}\right.$$

where $x_{i}$ is the actual value of each factor, $e_{i}$ is the upper and lower limits of the evaluation set.

The main disasters in Rongjiang County include landslide, collapse and debris flow. We selected a debris flow ditch in Rongjiang County as an example of the model application, and the indexes are shown in Table 4.

$$\begin{array}{c} R_{C_{1}}=\left[\begin{array}{cccc} 0 & 0 & 0 & 1\\ 0.075 & 0.925 & 0 & 0\\ 0 & 1 & 0 & 0\\ 0 & 0 & 0.333 & 0.667\\ 0 & 0 & 0.04 & 0.96 \end{array}\right]\\ R_{C_{2}}=\left[\begin{array}{cccc} 0 & 0 & 0 & 1\\ 0 & 0 & 0.14 & 0.86\\ 0 & 0 & 0.333 & 0.667 \end{array}\right]\\ R_{C_{3}}=\left[\begin{array}{cccc} 0 & 0 & 0 & 1\\ 0 & 0 & 1 & 0\\ 0 & 1 & 0 & 0 \end{array}\right] \end{array}$$

According to the weight vector *W* and fuzzy relation matrix *R*, the risk of debris flow can be evaluated by the fuzzy comprehensive method.

(9) $$O=\left\{b_{1},b_{2}\cdots b_{n}\right\}$$

(10) O=W×R

where O represents a fuzzy comprehensive evaluation set [33], W is the weight vector, R is the fuzzy relation matrix calculated according to the membership function.

The first-class fuzzy comprehensive evaluation can be obtained:

$$\begin{array}{c} W_{C_{1}}=(0.254,0.107,0.163,0.326,0.150)\\ W_{C_{2}}=(0.312,0.490,0.198)\\ W_{C_{3}}=(0.525,0.324,0.141)\\ O_{C_{1}}=W_{C_{1}}\cdot R_{C_{1}}=\\ (0.254,0.107,0.163,0.326,0.150)\left[\begin{array}{cccc} 0 & 0 & 0 & 1\\ 0.075 & 0.925 & 0 & 0\\ 0 & 1 & 0 & 0\\ 0 & 0 & 0.333 & 0.667\\ 0 & 0 & 0.04 & 0.96 \end{array}\right]=\\ \left(0.008,0.262,0.115,0.615\right) \end{array}$$

$$\begin{array}{c} O_{C_{2}}=W_{C_{2}}\cdot R_{C_{2}}=\\ (0.312,0.490,0.198)\left[\begin{array}{cccc} 0 & 0 & 0 & 1\\ 0 & 0 & 0.14 & 0.86\\ 0 & 0 & 0.333 & 0.667 \end{array}\right]=\\ \left(0,0.198,0.135,0.865\right) \end{array}$$

$$\begin{array}{c} O_{C_{3}}=W_{C_{3}}\cdot R_{C_{3}}=\\ (0.525,0.334,0.141)\left[\begin{array}{cccc} 0 & 0 & 0 & 1\\ 0 & 0 & 1 & 0\\ 0 & 1 & 0 & 0 \end{array}\right]=\\ \left(0,0.141,0.334,0.525\right) \end{array}$$

where $W_{C_{1}},W_{C_{2}},W_{C_{3}}$ is the weight vector of factor sets $C_{1},C_{2},C_{3}$, $O_{c}$ is the first-order fuzzy comprehensive evaluation vector.

The two-level fuzzy comprehensive evaluation:

$$\begin{array}{c} O_{V}=W_{V}\cdot \left[\begin{array}{l} O_{C_{1}}\\ O_{C_{2}}\\ O_{C_{3}} \end{array}\right]=\\ (0.549,0.240,0.201)\left[\begin{array}{cccc} 0.008 & 0.262 & 0.115 & 0.615\\ 0 & 0.195 & 0.135 & 0.865\\ 0 & 0.141 & 0.334 & 0.525 \end{array}\right]\\ =(0.004,0.219,0.163,0.651) \end{array}$$

Normalized result,

$$O_{V}=(0.057,0.311,0.231,0.922)$$

where $O_{V}$ is the second-order fuzzy comprehensive evaluation vector.

According to the principle of maximum membership degree, the highest value in the evaluation results is 0.922, which is in extreme risk. The results are in good agreement with the actual situation in this paper.

## 5. Conclusions

This study discusses the causes of the derailment accident of the D2809 train in Guizhou on 4 June 2022 and some suggestions are put forward to prevent train derailments caused by debris flow. The main conclusions are as follows:

(1) The train accident was mainly due to the local continuous rain and short-term heavy rain. During the high-speed operation, the train hit a sudden landslide that invaded the line, resulting in the derailment of the No. 7 and No. 8 train.

(2) The railway department immediately launched an emergency plan after the accident. The injured were sent to Rongjiang County hospital for all-out treatment, given priority to live. People used the golden time of rescue, responsible for the safety of the victims, and reduced the number of casualties to the minimum.

(3) The reduction of soil shear strength is the main reason for debris flow. In addition, flood control and landslide prevention are complicated during the rainy season in the mountainous areas in the south and southwest China. In order to prevent train accidents caused by debris flow, we must ensure strict investigation and site selection firstly. It is also essential to install protective retaining walls on high mountain slopes, establish a timely and effective debris flow early warning mechanism and reinforce the soil beside the tunnel entrance.

(4) This paper puts forward a risk prediction model of train debris flow derailment based on the fuzzy comprehensive evaluation method, and the debris flow valley near the incident site is selected for model verification. The results are in good agreement with the actual situation.

## Figures and Tables

**Table 2:** Classification standard on evaluation index of debris flow risk.

Evaluation Index	Low Risk (V_1_)	Moderate Risk (V_2_)	High Risk (V_3_)	Extreme Risk (V_4_)
Dully cross section	Flat type	Compound section	U-shaped valley	V-shaped and U-shaped middle valley
Main gully length (m)	≤1	1–5	5–10	≥10
Bad geological phenomena	None	Slight	Moderate	Serious
Longitudinal slope of main gully (°)	≤5	5–15	15–30	≥30
Maximum elevation difference of river basin (m)	0.2	0.2–0.5	0.5–1	≥1
Loose solid material reserves (10^4^ m^3^)	1	1–5	5–10	10
Maximum rainfall within 24 h (mm)	≤25	25–50	50–100	≥100
Vegetation coverage (%)	>60	30–60	10–30	<10
Protective facilities	Adequate	Common	Few	None
Soil reinforcement	Ade-quate	Common	Few	None
Importance of high-speed rail lines	Common	Important	Very important	Extreme important

**Table 3:** Evaluation factor weight value.

Category	Secondary Weight	Factor	First-Order Weight
		Dully cross section	0.254
		Main gully length	0.107
C1	0.549	Bad geological phenomena	0.163
		Longitudinal slope of main gully	0.326
		Maximum elevation difference of river basin	0.150
		Loose solid material reserves	0.312
C2	0.240	Maximum rainfall within 24 h	0.490
		Vegetation coverage	0.198
		Protective facilities	0.525
C3	0.201	Soil reinforcement	0.334
		Importance of high-speed rail lines	0.141

**Table 4:** Actual value of the evaluation factor.

Evaluation Index	D_1_	D_2_ (m)	D_3_	D_4_ (°)	D_5_ (m)	D_6_ (10^4^ m^3^)	D_7_(mm)	D_8_(%)	D_9_	D_10_	D_11_
Actual value	V shaped	4.7	slight	25	0.98	14.2	93.2	50	none	few	Important

## Data Availability

All data collected during the study are available in the submitted article and the detailed data values are available from the corresponding author by request.
